# The tumour-suppressive function of *miR-1* and *miR-133a* targeting TAGLN2 in bladder cancer

**DOI:** 10.1038/bjc.2011.23

**Published:** 2011-02-08

**Authors:** H Yoshino, T Chiyomaru, H Enokida, K Kawakami, S Tatarano, K Nishiyama, N Nohata, N Seki, M Nakagawa

**Affiliations:** 1 Department of Urology, Graduate School of Medical and Dental Sciences, Kagoshima University, 8-35-1 Sakuragaoka, Kagoshima 890-8520, Japan; 2 Department of Functional Genomics, Graduate School of Medicine, Chiba University, 1-8-1 Inohana, Chuo-ku, Chiba 260-8670, Japan

**Keywords:** TAGLN2, microRNA, *miR-1*, *miR-133a*, bladder cancer

## Abstract

**Background:**

On the base of the microRNA (miRNA) expression signature of bladder cancer (BC), we found that *miR-1* and *miR-133a* were significantly downregulated in BC. In this study, we focussed on the functional significance of *miR-1* and *miR-133a* in BC cell lines and identified a molecular network of these miRNAs.

**Methods and results:**

We investigated the miRNA expression signature of BC clinical specimens and identified several downregulated miRNAs (*miR-133a*, *miR-204*, *miR-1*, *miR-139-5p*, and *miR-370*). *MiR-1* and *miR-133a* showed potential role of tumour suppressors by functional analyses of BC cells such as cell proliferation, apoptosis, migration, and invasion assays. Molecular target searches of these miRNAs showed that *transgelin 2* (*TAGLN2*) was directly regulated by both *miR-1* and *miR-133a*. Silencing of TAGLN2 study demonstrated significant inhibitions of cell proliferation and increase of apoptosis in BC cell lines. The immunohistochemistry showed a positive correlation between TAGLN2 expression and tumour grade in clinical BC specimens.

**Conclusions:**

The downregulation of *miR-1* and *miR-133a* was a frequent event in BC, and these miRNAs were recognised as tumour suppressive. *TAGLN2* may be a target of both miRNAs and had a potential oncogenic function. Therefore, novel molecular networks provided by miRNAs may provide new insights into the underlying molecular mechanisms of BC.

Bladder cancer (BC) is the fourth most common tumour diagnosed and the second most common cause of death in patients with genitourinary tract malignancies worldwide (Parkin *et al*, 2005; Jemal *et al*, 2010). In Japan, the age-standardised mortality rate of BC has remained relatively stable in men but has increased slightly since 1993 in women (Qiu *et al*, 2009). There have been significant advances in treatment, including surgical techniques and adjuvant chemotherapy; however, BC continues to be a common disease with high mortality (Shirodkar and Lokeshwar, 2009). Therefore, new treatment modalities based on novel molecular networks in BC are desired.

MicroRNAs (miRNAs) are a class of small non-coding RNA molecules of 20–22 nucleotides that have a critical role in a variety of biological processes including development, differentiation, apoptosis, and cell proliferation. They regulate gene expression through translational repression and mRNA degradation. Although their biological functions remain largely unknown, recent studies suggest that miRNAs contribute to the development of various types of cancer (Ryan *et al*, 2010). A growing body of evidence indicates that miRNAs are aberrantly expressed in many human cancers, and they may function as oncogenes and tumour suppressors. Upregulated miRNAs could function as oncogenes by negatively regulating tumour-suppressor genes, whereas downregulated miRNAs could act as tumour suppressors, inhibiting cancers by regulating oncogenes (Lu *et al*, 2005; Calin and Croce, 2006; Ambs *et al*, 2008; Childs *et al*, 2009). Bioinformatic predictions indicate that miRNAs regulate >30% of the protein coding genes (Filipowicz *et al*, 2008). It is estimated that ∼1000 miRNAs exist in the vertebrate genome. So far, 1048 human miRNAs are registered at miRBase release 16.0 (http://microrna.sanger.ac.uk/).

*MiR-1* and *miR-133a* were among the top five downregulated miRNAs in our screening. *MiR-1* and *miR-133a* are muscle-enriched miRNAs that inhibit proliferation of progenitor cells and promote myogenesis by targeting *histone deacetylase* (*HDAC4*) and *serum response factor* (*SRF*), respectively (Chen *et al*, 2006). Recently, m*iR-1* and m*iR-133a* have been reported to be downregulated in various cancers and to have tumour-suppressive functions (Datta *et al*, 2008; Nasser *et al*, 2008; Yan *et al*, 2009; Uchida *et al*, 2010; Chiyomaru *et al*, 2010a). We have reported that *miR-133a* directly regulated oncogenic *FSCN1*, *LASP1*, and *GSTP1* genes in human BC (Uchida *et al*, 2010; Chiyomaru *et al*, 2010a, 2010b). We recognised that *miR-1* and *miR-133a* are located on the same chromosomal loci (18q11.2 and 20q13.33) (Chiyomaru *et al*, 2010b). Like this, several miRNAs are located on the same chromosomal region, in a so-called ‘cluster’. Recent studies demonstrated that *miR-17-92* cluster and *miR-221/222* cluster, which harbour oncogenic miRNAs, had important roles in several human malignancies (Zhang *et al*, 2009; Di Leva *et al*, 2010). However, the functional roles of the target genes regulated by these miRNA clusters have not been thoroughly investigated.

The aim of this study was to investigate whether *miR-1* has tumour-suppressive function in BC cell lines and to find common target genes of *miR-1* and *miR-133a*. We focussed on *transgelin 2* (*TAGLN2*), which was one of the most downregulated genes in oligo-microarray studies using an *miR-1* transfectant and is a putative target gene of *miR-1* and *miR-133a* as suggested by web-based software. The functional role of TAGLN2 has not yet been determined. Previous studies have reported that high expressions of TAGLN2 were observed in various human malignancies (Chen *et al*, 2005; Shi *et al*, 2005; Huang *et al*, 2006; Rho *et al*, 2009; Zhang *et al*, 2010). We hypothesised that *miR-1* and *miR-133a* directly regulate *TAGLN2*, which may have an oncogenic function in BC. We performed a luciferase reporter assay to determine whether *TAGLN2* mRNA is actually targeted by *miR-1* and *miR-133a* and a loss-of-function study using BC cell lines to investigate the functional roles of TAGLN2 in BC.

## Materials and methods

### Clinical specimens and cell culture

Tissue specimens for miRNA screening using a low-density array (LDA) were from 11 BC patients who had undergone cystectomy or transurethral resection of bladder tumours (TUR-BT) at Kagoshima University Hospital between 2007 and 2008. The tissue specimens for quantitative RT–PCR were from 23 BC patients who had received cystectomy or TUR-BT at Kagoshima University Hospital between 2006 and 2009. The patients’ backgrounds and clinicopathological characteristics are summarised in Supplementary Table 1. Normal bladder epitheliums (NBEs) were derived from patients with noncancerous disease. These specimens were staged according to the American Joint Committee on Cancer/Union Internationale Contre le Cancer tumour-node-metastasis classification and histologically graded (Sobin and Wittekind, 2002). Our study was approved by the Bioethics Committee of Kagoshima University; written previous informed consent and approval were given by these patients.

We used two human BC cell lines: BOY, which was established in our laboratory from an Asian male patient aged 66 years who was diagnosed with stage III BC with lung metastasis; and T24, which was invasive and obtained from the American Type Culture Collection. These cell lines were maintained in a minimum essential medium (MEM) supplemented with 10% fetal bovine serum in a humidified atmosphere of 5% CO_2_ and 95% air at 37 °C.

### Tissue collection and RNA extraction

Tissues were immersed in RNAlater (QIAGEN, Valencia, CA, USA) and stored at −20 °C until the RNA extraction. Total RNA including miRNA was extracted using the mirVana miRNA isolation kit (Ambion, Austin, TX, USA) following the manufacturer's protocol. The integrity of the RNA was checked with RNA 6000 Nano Assay Kit and a 2100 Bioanalyzer (Agilent Technologies, Santa Clara, CA, USA).

### MiRNA expression signatures and data normalisation

MicroRNA expression patterns were evaluated using the TaqMan LDA Human microRNA Panel v2.0 (Applied Biosystems, Foster City, CA, USA). The assay was composed of two steps: generation of cDNA by reverse transcription and a TaqMan real-time PCR assay. The description of real-time PCR and the list of human miRNAs can be found on the company’s website (http://www.appliedbiosystems.com). An analysis of relative miRNA expression data was performed using GeneSpring GX version 7.3.1 software (Agilent Technologies) according to the manufacturer’s instructions. A cutoff *P-*value of <0.05 was used to narrow down the candidates after global normalisation of the raw data. After global normalisation, additional normalisation was done by *RNU48* and *MammU6*.

### Quantitative real-time RT–PCR

TaqMan probes and primers for TAGLN2 (P/N: Hs00761239_m1; Applied Biosystems) were assay-on-demand gene expression products. All reactions were performed in duplicate and a negative control lacking cDNA was included. We followed the manufacturer's protocol for PCR conditions. Stem-loop RT–PCR (TaqMan MicroRNA Assays; P/N: PM10617 for *miR-1*, and PM10413 for *miR-133a*; Applied Biosystems) was used to quantitate miRNAs according to the earlier published conditions (Ichimi *et al*, 2009). To normalise the data for quantification of *TAGLN2* mRNA and the miRNAs, we used *human GUSB* (P/N: Hs99999908_m1; Applied Biosystems) and *RNU48* (P/N: 001006; Applied Biosystems), respectively, and the ΔΔCt method was employed to calculate the fold change. As a control RNA, we used Premium Total RNA from normal human bladder (AM7990; Applied Biosystems).

### Mature miRNA and siRNA transfection

As described elsewhere (Ichimi *et al*, 2009), the BC cell lines were transfected with Lipofectamine RNAiMAX transfection reagent (Invitrogen, Carlsbad, CA, USA) and Opti-MEM (Invitrogen) with 10 nM of mature miRNA molecules. Pre-miR and negative-control miRNA (Applied Biosystems) were used in the gain-of-function experiments, whereas *TAGLN2* siRNA (Cat no. HSS144745 and HSS144746; Invitrogen) and negative-control siRNA (D-001810-10; Thermo Fisher Scientific, Waltham, MA, USA) were used in the loss-of-function experiments. Cells were seeded in a 10-cm dish for protein extraction (8 × 10^5^ per dish), in a six-well plate for apoptosis (10 × 10^4^ per well) and for wound healing assay (20 × 10^4^ per well), in a 24-well plate for mRNA extraction and luciferase reporter assay (5 × 10^4^ per well), and in a 96-well plate for XTT assay (3000 per well).

### Cell proliferation, migration, and invasion assays

Cell proliferation was determined using an XTT assay (Roche Applied Sciences, Tokyo, Japan) performed according to the manufacturer’s instructions. Cell migration activity was evaluated by wound healing assay. Cells were plated in six-well dishes, and the cell monolayer was scraped using a P-20 micropipette tip. The initial gap length (0 h) and the residual gap length 24 h after wounding were calculated from photomicrographs. A cell invasion assay was carried out using modified Boyden Chambers consisting of transwell-precoated matrigel membrane filter inserts with 8-mm pores in 24-well tissue culture plates (BD Biosciences, Bedfold, MA, USA). Minimum essential medium containing 10% fetal bovine serum in the lower chamber served as the chemoattractant as described previously (Chiyomaru *et al*, 2010a). All experiments were performed in triplicate.

### Apoptosis analysis

The BC cell lines transiently transfected with transfection reagent only (mock), si-control, si-TAGLN2, miR-control, *miR-1*, or *miR-133a* in six-well tissue culture plates as described earlier were harvested 72 h after transfection by trypsinisation and washed in cold PBS. Double staining with FITC-Annexin V and propidium iodide (PI) was carried out using the FITC Annexin V Apoptosis Detection Kit (BD Biosciences) according to the manufacturer's recommendations and immediately analysed within an hour by flow cytometry (FACScan; BD Biosciences). Cells were discriminated into viable cells, dead cells, early apoptotic cells, and apoptotic cells by the CellQuest software (BD Biosciences), and then the percentages of early apoptotic and apoptotic cells from each experiment were compared. Experiments were done in triplicate.

### Target gene search for *miR-1*

Oligo-microarray Human 44K (Agilent) was used for expression profiling in *miR-1*-transfected BC cell lines (BOY and T24) in comparison with miR-negative control transfectant, as previously described (Chiyomaru *et al*, 2010a). Briefly, hybridisation and washing steps were performed in accordance with the manufacturer’s instructions. The arrays were scanned using a Packard GSI Lumonics ScanArray 4000 (PerkinElmer, Boston, MA, USA). The data obtained were analysed with DNASIS array software (Hitachi Software Engineering, Tokyo, Japan), which converted the signal intensity for each spot into text format. The Log2 ratios of the median subtracted background intensity were analysed. Data from each microarray study were normalised by global normalisation.

The predicted target genes and their miRNA binding site seed regions were investigated using TargetScan (release 5.1, http://www.targetscan.org/). The sequences of the predicted mature miRNAs were confirmed using miRBase (release 16.0, September 2010; http://microrna.sanger.ac.uk/).

### Western blots

After 3 days of transfection, protein lysate (20 *μ*g) was separated by NuPAGE on 4–12% bis-tris gel (Invitrogen) and transferred into a polyvinylidene fluoride membrane. Immunoblotting was done with diluted (1 : 150) polyclonal TAGLN2 antibody (HPA001925; Sigma-Aldrich, St Louis, MO, USA) and GAPDH antibody (MAB374; Chemicon, Temecula, CA, USA). The membrane was washed and then incubated with goat anti-rabbit IgG (H+L)-HRP conjugate (Bio-Rad, Hercules, CA, USA). Specific complexes were visualised with an echochemiluminescence (ECL) detection system (GE Healthcare, Little Chalfont, UK), and the expression level of these genes was evaluated using ImageJ software (ver. 1.43; http://rsbweb.nih.gov/ij/index.html).

### Plasmid construction and dual-luciferase reporter assay

The miRNA target sequences were inserted between the *Xho*I–*Pme*I restriction sites in the 3′UTR of the *hRluc* gene in the psiCHECK-2 vector (C8021; Promega, Madison, WI, USA). Primer sequences for full-length 3′UTR of *TAGLN2* mRNA (5′-ATCGCTCGAGACAGATGGGCACCAACCGCG-3′ and 5′-CTCTAGGTTTAAACATCTTCCTCAAGCCCCAGAC-3′) were designed. Specific miRNA target sequences (40 bp length, Supplementary Table 2) for *miR-1* and *miR-133a* were artificially synthesised and inserted in the vector. Following that, T24 cells were transfected with 15 ng of vector, 10 nM of miRNA, and 1 *μ*l of Lipofectamine 2000 (Invitrogen) in 100 *μ*l of Opti-MEM (Invitrogen). The activities of firefly and *Renilla* luciferases in cell lysates were determined with a dual-luciferase assay system (E1910; Promega). Normalised data were calculated as the quotient of *Renilla*/firefly luciferase activities.

### Immunohistochemistry

A tissue microarray of 47 urothelial carcinomas and 8 normal bladders was obtained from US Biomax, Inc. (BL208; Rockville, MD, USA). Detailed information on all tumour specimens can be found at http://www.biomax.us/index.php. Immunostaining was done on the tissue microarray following the manufacturer's protocol. The primary rabbit polyclonal antibodies against TAGLN2 (Sigma-Aldrich) were diluted by 1 : 25. The slides were treated with biotinylated anti-rabbit IgG (H+L) made in goat (Vector Laboratories, Burlingame, CA, USA). Diaminobenzidine-hydrogen peroxide (Sigma-Aldrich) was the chromogen, and the counterstaining was done with 0.5% haematoxylin. The positivity of endothelia served as an inner positive control. Immunostaining was evaluated according to a scoring method as described previously (Zhang *et al*, 2010). Each case was scored on the basis of the intensity and area of staining. The intensity of staining was graded on the following scale: 0, no staining; 1+, mild staining; 2+, moderate staining; and 3+, intense staining. The area of staining was evaluated as follows: 0, no staining of cells in any microscopic fields; 1+, <30% of cells stained positive; 2+, 30–60% stained positive; and 3+, >60% stained positive. A combined staining score (intensity+extension) of ⩽2 was low expression, a score between 3 and 4 was moderate expression, and a score between 5 and 6 was high expression.

### Statistical analysis

The relationship between two variables and the numerical values obtained by real-time RT–PCR was analysed using the Mann–Whitney *U*-test. The relationship among three variables and the numerical values was analysed using the Bonferroni-adjusted Mann–Whitney *U*-test. The *χ*^2^-test was used to evaluate the relationships between the immunohistochemical score of TAGLN2 expression and clinicopathological factors. Expert StatView analysis software (version 4; SAS Institute Inc., Cary, NC, USA) was used in both cases. In the comparison among three variables, a nonadjusted statistical level of significance of *P*<0.05 corresponds to a Bonferroni-adjusted level of *P*<0.0167.

## Results

### Identification of downregulated miRNAs in BC by miRNA expression signatures

We evaluated mature miRNA expression levels of 11 BC and 5 NBE specimens by miRNA expression signatures. A total of 41 and 19 downregulated miRNAs were selected after the normalisation using *RNU48* and *MammU6*, respectively (Supplementary Tables 3 and 4). The 17 miRNAs were commonly downregulated with *RNU48* and *MammU6* normalisation (Table 1). The top five miRNAs (*miR-133a*, *miR-204*, *miR-1*, *miR-139-5p*, and *miR-370*) in the list were subjected to further study.

### Detection of *miR-133a*, *miR-204*, *miR-1*, *miR-139-5p*, and *miR-370* expression by quantitative stem-loop RT–PCR

**Figure 1:**
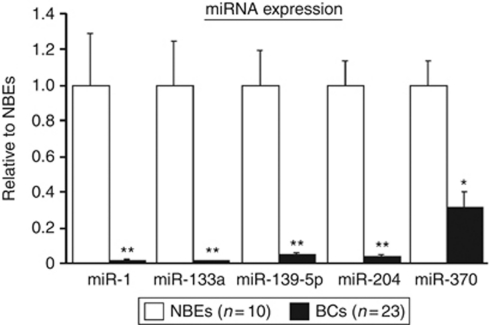
MicroRNA expression levels in clinical specimens. Real-time RT–PCR showed that miRNA expression in BCs was lower than that of NBEs. ^*^P<0.005; ^**^P<0.0001.

**Figure 2:**
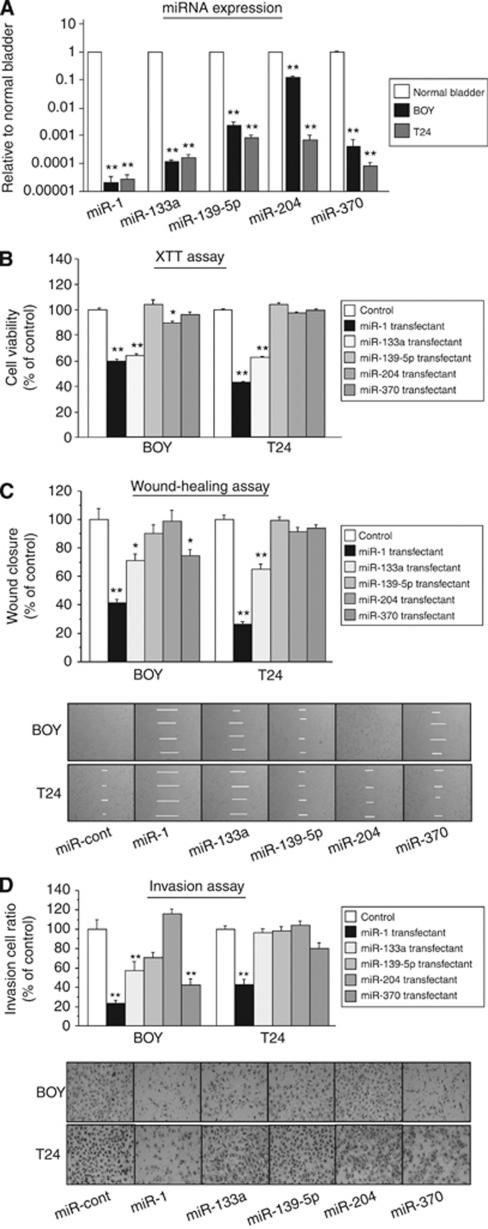
(**A**) MicroRNA expression levels in BC cell lines. Real-time RT–PCR showed that miRNA expression in BC cell lines (BOY and T24) was lower than that of the normal human bladder RNA. (**B**–**D**) Effect of cell viabilities in miRNA (*miR-1, miR-133a, miR-139-5p, miR-204*, *and miR-370*) transfectants: (**B**) cell proliferation determined by the XTT assay; (**C**) cell migration activity determined by the wound healing assay; and (**D**) cell invasion activity determined by the matrigel invasion assay in BOY and T24 cell lines transfected with the miRNAs. ^*^P<0.005; ^**^P<0.0001.

Quantitative stem-loop RT–PCR demonstrated that the expression levels of the top five miRNAs (*miR-133a*, *miR-204*, *miR-1*, *miR-139-5p*, and *miR-370*) were significantly lower in 23 BC specimens in comparison with 10 NBEs (*P*<0.005; Figure 1). These miRNA expressions were significantly lower in BC cell lines (BOY and T24) in comparison with the normal human bladder RNA (*P*<0.0001; Figure 2A).

### Effect of the downregulated miRNA transfection on cell proliferation, migration activity, and invasion in BC cell lines

To investigate the functional role of the five selected miRNAs, we performed gain-of-function studies using the miRNA transfectants. The XTT assay showed significant cell proliferation inhibitions in *miR-1* and *miR-133a* transfectants in comparison with the miR-control transfectants (percentage of cell viability for BOY: 59.9±1.4, 64.1±1.4, and 100.0±1.2, respectively, *P*<0.0001; and for T24: 43.3±0.6, 62.4±0.7, and 100.0±0.7, respectively, *P*<0.0001), but no significant inhibition was observed in other miRNA transfectants except the miR-204-transfected BOY cell line (Figure 2B). The wound healing assay showed significant cell migration inhibitions in *miR-1* and *miR-133a* transfectants in comparison with the controls (percentage of wound closure for BOY; 41.4±2.5, 71.3±4.4, and 100.0±3.2, respectively, *P*<0.0001; and for T24: 26.0±2.4, 65.2±3.9, and 100.0±3.2, respectively, *P*<0.0001), but no significant inhibition was observed in other miRNA transfectants except *miR-370*-transfected BOY cell line (Figure 2C). Matrigel invasion assay demonstrated that invading cell numbers were significantly decreased in both *miR-1-* and *miR-133a*-transfected BOY cell lines and *miR-1-*transfected T24 cell line in comparison with the controls (percentage of cell invasion for BOY: 23.4±3.0, 57.3±8.9, and 100.0±10.0, respectively, *P*<0.0001; and for T24: 43.4±4.8, 96.5±4.4, and 100.0±3.8, respectively, *P*<0.0001; the *miR-1* transfectant *vs* control), but no significant inhibition was observed in other miRNA transfectants except the miR-370-transfected BOY cell line (Figure 2D). To evaluate the simultaneous effect of miR-1 and miR-133a, we evaluated another XTT assay by using *miR-1* and *miR-133a* co-transfected BOY and T24 cell lines. We found similar effects of cell viability inhibition by the transfectants in comparison with that of each *miR-1* or *miR-133a* transfectants (Supplementary Figure 1). Because *miR-1* and *miR-133a* transfection had a significantly stronger tumour-suppressive effect among the five miRNAs, they were subjected to further analyses as the leading candidates for tumour-suppressive miRNAs in BC.

### *MiR-1* induced apoptosis in BC cell lines

**Figure 3:**
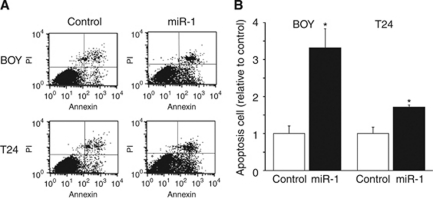
(**A**) Apoptosis assay determined by flow cytometry. Early apoptotic cells can be seen in the bottom right quadrant and late are in the upper right. (**B**) The normalised ratio of the apoptosis assay is shown in the histogram. ^*^P<0.05.

Cell apoptosis in *miR-1* transfectants was detected using flow cytometry. As shown by representative images in Figure 3A, the apoptotic cell fractions (early apoptotic and apoptotic; lower right and upper right, respectively) were greater in *miR-1* transfectants than in miR-control transfectants (BOY and T24). As shown in Figure 3B, *miR-1* transfection induced apoptosis in BC cell lines (BOY, 3.31±0.51 and 1.00±0.20; T24, 1.70±0.08 and 1.00±0.17, respectively, *P*<0.05). In terms of *miR-133a*, we previously demonstrated that *miR-133a* transfection also induced apoptosis in the same BC cell lines (Uchida *et al*, 2010).

### Gene expression profile identifying downregulated genes in *miR-1* transfectant

To gain further insight into which genes are affected by *miR-1* transfection, we performed gene expression analysis with *miR-1* transfectants and the controls (BOY and T24 cells). A total of 18 genes were downregulated less than –4.0-fold in *miR-1* transfectants compared with the controls (Table 2). The TargetScan program showed that 17 genes had putative target sites of *miR-1* in their 3′UTR (Table 2). Previously, we had performed gene expression analysis with *miR-133a* transfectants (Uchida *et al*, 2010), and the *TAGLN2* gene was commonly listed in the top 10 downregulated genes in current (*miR-1*) and former (*miR-133a*) signatures. Therefore, we focussed on the *TAGLN2* gene as a promising candidate targeted by both *miR-1* and *miR-133a*. Entries from the former and the current microarray data were approved by the Gene Expression Omnibus (GEO) and were assigned GEO accession numbers GSE19717 and GSE24782.

### TAGLN2 expression in BC cell lines and TAGLN2 silencing by *miR-1* and *miR-133a* transfection

**Figure 4:**
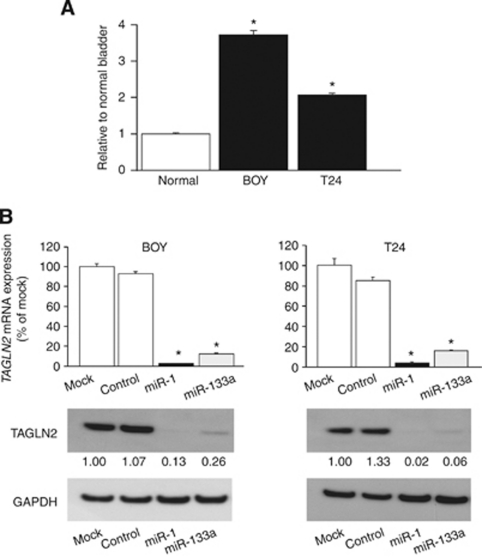
(**A**) The mRNA expression of *TAGLN2* in the BOY and T24 cell lines and the normal human bladder RNA. The mRNA expression of *TAGLN2* was more than two-fold in BC cell lines compared with the normal human bladder RNA. (**B**, upper) *TAGLN2* mRNA expression after 24 h of transfection with 10 nM of the miRNA (*miR-1* and *miR-133a*). (**B**, lower) TAGLN2 protein expression after 72 h of transfection of miRNA. GAPDH was used as a loading control. The protein expression level of TAGLN2 was also repressed in the transfectants.

The quantitative real-time RT–PCR analysis showed that the mRNA expression of *TAGLN2* in the BOY and T24 cell lines was more than two-fold higher than that in the normal human bladder RNA (Figure 4A). To examine the functional role of TAGLN2, we performed gain-of-function studies using *miR-1* and *miR-133a* transfectant (BOY and T24) cell lines, and the mRNA and protein expression levels of TAGLN2 were markedly downregulated in the transfectants in comparison with the controls (Figure 4B).

### TAGLN2 as a target of post-transcriptional repression by *miR-1* and *miR-133a*

**Figure 5:**
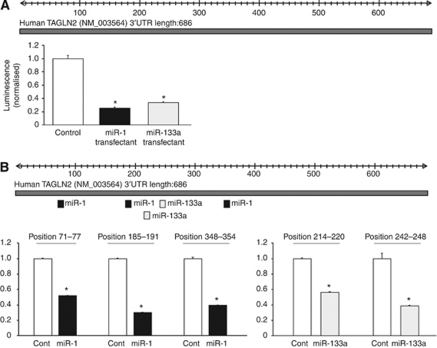
*MiR-1* and *miR-133a* binding sites in 3′-UTR of *TAGLN2* mRNA. (**A**) A luciferase reporter assay using the vector encoding full-length 3′-UTR of *TAGLN2* mRNA. The *Renilla* luciferase values were normalised by firefly luciferase values. (**B**) Luciferase reporter assays using the vectors encoding putative target sites of TAGLN2 3′-UTR: three target sites for *miR-1* and two sites for *miR-133a*.

We performed a luciferase reporter assay to determine whether *TAGLN2* mRNA has a target site for *miR-1* and *miR-133a.* We used a vector encoding full-length 3′UTR of *TAGLN2* mRNA and found that the luminescence intensity was significantly reduced in the *miR-1* and *miR-133a* transfectant (Figure 5A). Furthermore, the luminescence intensity significantly decreased at the three sites targeted by *miR-1* (position 71–77, 185–191, and 348–354) and two sites targeted by *miR-133a* (position 214–220 and 242–248) (Figure 5B).

### Effect of TAGLN2 knockdown on cell proliferation, invasion, and migration activity in BC cell lines

**Figure 6:**
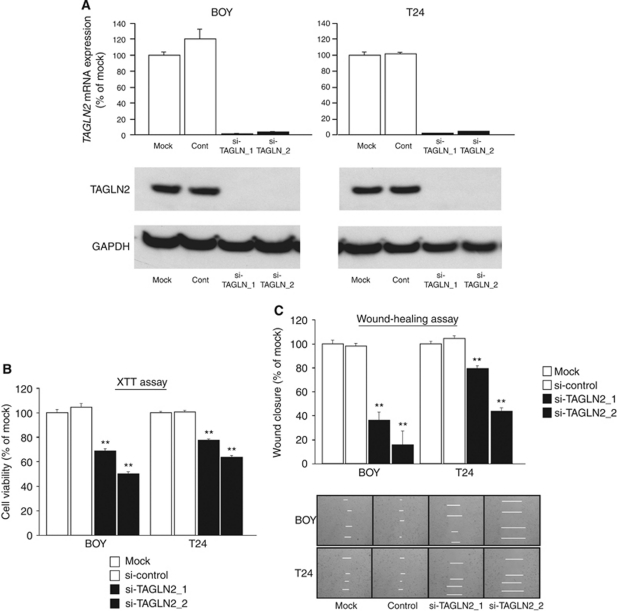

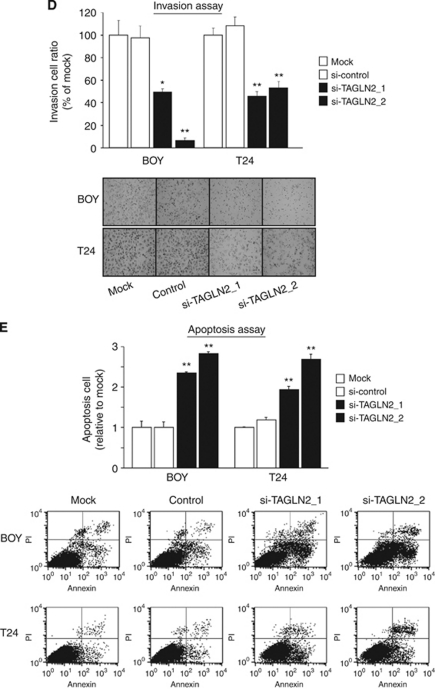
(**A**, upper) *TAGLN2* mRNA expression after 24 h of transfection with 10 nM of si-TAGLN2. *TAGLN2* mRNA expression was repressed in si-TAGLN2 transfectants. (**A**, lower) TAGLN2 protein expression after 72 h of transfection of the siRNAs. GAPDH was used a loading control. (**B**–**D**) TAGLN2-knockdown effects on BC cell viability by si-RNA. (**B**) Cell proliferation determined by the XTT assay; (**C**) cell migration activity determined by the wound healing assay; and (**D**) cell invasion activity determined by the matrigel invasion assay in BOY and T24 cell lines transfected with si-TAGLN2. ^*^P<0.0005; ^**^P<0.0001. (**E**) Apoptosis assay determined by flow cytometry. Early apoptotic cells can be seen in the bottom right quadrant and late are in the upper right. The normalised ratio of the apoptosis assay is shown in the histogram. ^**^P<0.0001.

To examine the functional role of TAGLN2, we performed loss-of-function studies using two different si-TAGLN2 transfections into BOY and T24 cell lines. The mRNA and protein expression of TAGLN2 was markedly repressed by these si-TAGLN2 transfections (Figure 6A). The XTT assay revealed significant cell proliferation inhibition in the two si-TAGLN2 transfectants in comparison with that in the untransfectants (mock) and the si-control transfectants (percentage of cell viability for BOY: 69.0±1.3, 49.7±2.0, 100.0±2.6, and 104.6±2.9, respectively, *P*<0.0001; and for T24: 77.4±1.4, 63.6±1.4, 100.0±1.2, and 100.5±1.6, respectively, *P*<0.0001; Figure 6B). The wound healing assay also demonstrated significant cell migration inhibitions in the two si-TAGLN2 transfectants compared with the counterparts (percentage of wound closure for BOY: 36.0±7.3, 15.8±11.4, 100.0±3.0, and 98.2±2.6, respectively, *P*<0.0001; and for T24: 79.3±2.6, 43.6±3.2, 100.0±2.3, and 104.3±2.3, respectively, *P*<0.0001; Figure 6C). The matrigel invasion assay demonstrated that the number of invading cells was significantly decreased in the two si-TAGLN2 transfectants compared with the counterparts (percentage of cell invasion for BOY: 49.2±3.3, 6.2±2.7, 100.0±12.8, and 97.7±10.7, respectively, *P*<0.0005; and for T24: 45.7±4.0, 53.2±5.3, 100.0±6.0, and 108.3±7.6, respectively, *P*<0.0001; Figure 6D).

### TAGLN2 knockdown-induced apoptosis in BC cell lines

The apoptotic cell fractions were greater in the two si-TAGLN2 transfectants than those in the mock and si-control transfectant at 72 h after transfection (relative to mock; BOY: 2.34±0.04, 2.84±0.05, 1.00±0.15, and 1.00±0.15, respectively, *P*<0.0001; T24: 1.93±0.09, 2.70±0.12, 1.00±0.00, and 1.18±0.07, respectively, *P*<0.0001; Figure 6E).

### Immunohistochemistry of TAGLN2 in tissue microarray

**Figure 7:**
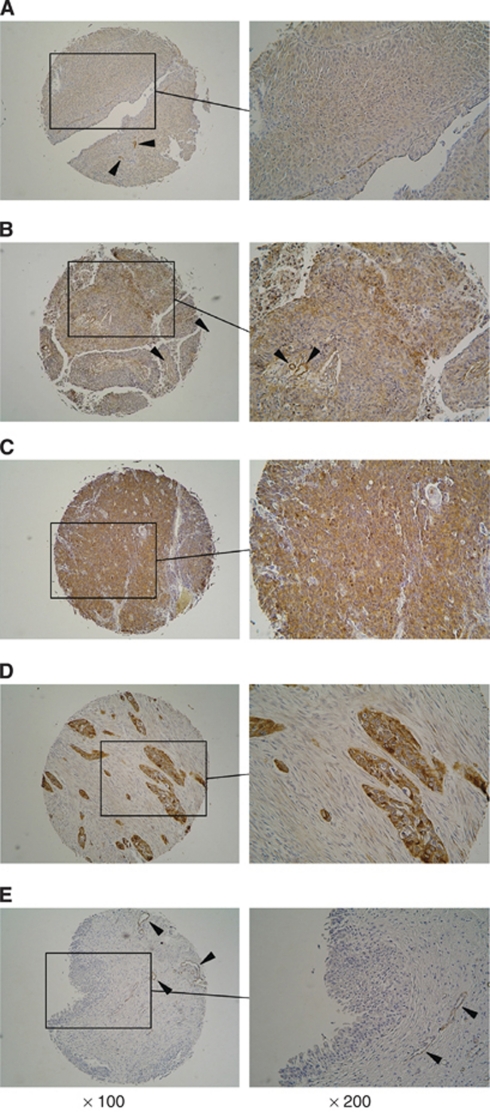
Immunohistochemical staining of TAGLN2 in tissue specimens. (**A**) Positively stained tumour lesion (Grade 1, T2bN0M0); (**B**) positively stained tumour lesion (Grade 2, T3N0M0); (**C**) positively stained tumour lesion (Grade 3, T2N0M0); (**D**) positive staining in metastatic BC cells of metastatic region (Grade 3); (**E**) negative staining in normal urocustis tissue. (**A–D**) Positive staining in tumour cells: weak (**A**), moderate (**B**), and strong (**C**, **D**). Some microvessel walls also stained positive for TAGLN2 (**A**, **B**, **E** arrowheads).

Figure 7 shows representative results of immmunohistochemical staining of TAGLN2. The TAGLN2 was strongly expressed in several tumour lesions: A (Grade 1, T2bN0M0), B (Grade 2, T3N0M0), C (Grade 3, T2N0M0), and D (Grade 3, metastatic region), whereas no expression was observed in the normal tissue (E); the expression score of tumours was significantly higher than that of normal tissues (*P*=0.0202). We found that there were significant correlations between the expression scores and tumour grade/metastasis (*P*=0.0148 and *P*=0.0145, respectively; Table 3).

## Discussion

In this study, we identified 17 downregulated miRNAs that survived after three different normalisation methods. Among them, we tested *miR-133a*, *miR-204*, *miR-1*, *miR-139-5p*, and *miR-370*, which were the top five downregulated miRNAs. Investigators had demonstrated that *miR-204* was a tumour-suppressive miRNA in head and neck tumour (Lee *et al*, 2010); *miR-139-5p* was downregulated in endometrial serous adenocarcinoma (Hiroki *et al*, 2010); and *miR-370* expression was silenced by promoter hypermethylation in malignant human cholangiocytes (Meng *et al*, 2008). The expression levels of the five miRNAs were indeed downregulated in BC specimens as well as in BC cell lines. However, we found typical tumour-suppressive effects in *miR-1*- and *miR-133a*-transfected BC cell lines, and these miRNAs were plausible to be critical for BC development.

The *miR-1* and *miR-133* are cardiac and skeletal muscle-specific, bicistronic miRNAs transcriptionally controlled by some major regulators of muscle differentiation (Chen *et al*, 2006; Williams *et al*, 2009). These miRNAs have been reported to be downregulated in human malignancies (Datta *et al*, 2008; Nasser *et al*, 2008; Yan *et al*, 2009; Uchida *et al*, 2010; Chiyomaru *et al*, 2010a). Ectopic expression of *miR-1* in HCC, lung cancer, prostate cancer, and rhabdomyosarcoma inhibited tumour cell growth (Ambs *et al*, 2008; Datta *et al*, 2008; Nasser *et al*, 2008; Yan *et al*, 2009). However, the function of *miR-1* in BC remains to be elucidated. *MiR-1* had an important role in the regulation of apoptosis, which is involved in post-transcriptional repression of BCL2 in cardiomyocyte (Tang *et al*, 2009). In cancer research fields, ectopic *miR-1* induced apoptosis through enhanced activation of caspases 3 and 7, cleavage of their substrate PARP-1, and depletion of antiapoptotic Mcl-1 in lung cancer cells (Nasser *et al*, 2008). Consistent with previous studies, re-expression of *miR-1* in BC cell lines resulted in induction of apoptosis and reduced cell viability in this study. Regarding *miR-133a*, previous studies demonstrated that its expression was downregulated in pancreatic ductal adenocarcinoma, oesophageal squamous cell carcinoma, rhabdomyosarcoma, colorectal cancer, and squamous cell carcinoma of the tongue (Bandrés *et al*, 2006; Szafranska *et al*, 2007; Wong *et al*, 2008a, 2008b; Arndt *et al*, 2009; Yan *et al*, 2009; Kano *et al*, 2010). *MiR-133a* inhibited proliferation and induced apoptosis and directly bound to pyruvate kinase type M2 expression, which are potent oncogenes (Wong *et al*, 2008b). However, there has been no study concerning genes targeted by both *miR-1* and *miR-133a*, which are clustered on the same chromosomal loci in human malignancies. In BC, we have reported that *miR-133a* had a critical role in regulating oncogenic FSCN1, LASP1, and GSTP1 (Uchida *et al*, 2010; Chiyomaru *et al*, 2010a, 2010b) and have demonstrated for the first time that LASP1 was the target of *miR-1*/*miR-133a* cluster (Chiyomaru *et al*, 2010b). In this study, the *TAGLN2* gene was found to be another target of the *miR-1*/*miR-133a* cluster. It is plausible that the *miR-1*/*miR-133a* cluster may have important roles as tumour suppressors through downregulating these oncogenic genes. However, we found no simultaneous effect of cell viability inhibition by *miR-1* and *miR-133a* co-transfection, suggesting that each miRNA may strongly repress same target genes and no additional effect might be caused by the other miRNA. Further investigations are necessary to elucidate the simultaneous effect of the *miR-1*/*miR-133a* cluster. Our data suggest that retrieved expression of *miR-1*/*miR-133a* clusters could be a new therapeutic strategy for BC.

*TAGLN2* contains a conserved actin-binding domain also known as the calponin (a calcium-binding protein) homologue domain. TAGLN and TAGLN3 are homologues of TAGLN2, and TAGLN3 is a novel neuron-specific protein and has not been reported in cancer (Ito *et al*, 2005). The protein encoded by the *TAGLN* gene is an actin-binding protein like TAGLN2, found in fibroblasts and smooth muscle. Overexpression of the TAGLN protein has been observed in carcinomas of the stomach, liver, and oesophagus (Rho *et al*, 2009). Although the function of TAGLN2 is unknown, there have been a number of reports concerning the relationship between TAGLN2 expression and tumourigenesis (Chen *et al*, 2005; Shi *et al*, 2005; Huang *et al*, 2006; Rho *et al*, 2009; Zhang *et al*, 2010). Overexpression of TAGLN2 was observed in HCC and pancreatic cancer (Chen *et al*, 2005; Shi *et al*, 2005; Huang *et al*, 2006). Zhang *et al* (2010) demonstrated that increased TAGLN2 expression was correlated with lymph node metastasis, distant metastasis, and the TNM classification in colorectal cancer. We also found a significant correlation of TAGLN2 expression with metastasis region and tumour grade despite of no correlation with tumour stage. Our tissue microarray included no Ta tumour and only four metastasis-positive patients. Studies for larger number of samples with balanced pathological background are needed to elucidate the precise correlation between TAGLN2 expression and clinicopathological parameters. These studies have implied that TAGLN2 may represent a potential tumour biomarker. We found that cell viability was markedly decreased in TAGLN2 knockdown cells by inducing apoptosis, which suggests that this molecule may have oncogenic function. However, it is still unknown how TAGLN2, which is an actin-binding protein, interacts with apoptosis. Further examinations are necessary to elucidate this.

In summary, *miR-1*/*miR-133a* clusters may function as tumour suppressors through repression of oncogenic TAGLN2 in BC. *MiR-1/miR-133a* transfection and TAGLN2 knockdown resulted in decreased BC cell viability and induction of apoptosis. Novel molecular networks provided by miRNAs may provide new insights into the underlying molecular mechanisms of BC.

## Figures and Tables

**Table 1:** Downregulated microRNAs in bladder cancer (BC)

				**Fold change**
**microRNA**	***P*-value**	**Normal**	**Cancer**	**Cancer/normal**
*hsa-miR-133a*	3.50E–02	1.17E–01	2.48E–03	2.12E–02
*hsa-miR-204*	4.50E–02	4.51E–03	2.08E–04	4.61E–02
*hsa-miR-1*	9.40E–03	1.52E–03	7.16E–05	4.72E–02
*hsa-miR-139-5p*	1.71E–04	8.31E–02	4.65E–03	5.60E–02
*hsa-miR-370*	2.37E–02	8.95E–04	8.26E–05	9.23E–02
*hsa-miR-133b*	3.60E–02	1.78E–03	1.26E–04	7.06E–02
*hsa-miR-574-3p*	6.11E–04	3.25E–01	3.63E–02	1.12E–01
*hsa-miR-376c*	2.57E–03	1.44E–02	1.91E–03	1.32E–01
*hsa-miR-214*	2.62E–03	5.08E–02	7.29E–03	1.43E–01
*hsa-let-7c*	1.34E–03	4.70E–03	7.16E–04	1.52E–01
*hsa-miR-140-3p*	6.29E–03	1.73E–02	2.96E–03	1.72E–01
*hsa-miR-134*	7.02E–04	3.54E–03	6.69E–04	1.89E–01
*hsa-miR-411*	1.43E–03	4.58E–03	1.05E–03	2.29E–01
*hsa-miR-218*	1.83E–03	1.66E–02	4.06E–03	2.44E–01
*hsa-miR-196b*	6.14E–03	2.76E–02	7.56E–03	2.74E–01
*hsa-miR-186*	8.04E–04	8.68E–02	3.10E–02	3.57E–01
*hsa-miR-320*	3.81E–02	2.34E–01	9.70E–02	4.14E–01

**Table 2:** Downregulated genes in *miR-1* transfectants

		**Fold change (log 2 ratio)**				
**Entrez gene ID**	**Symbol**	**BOY**	**T24**	**Average**	**Description**	**Target sites**
23446	*SLC44A1*	−3.86	−3.69	−3.77	*Solute carrier family 44, member 1*	+
114902	*C1QTNF5*	−3.92	−3.48	−3.70	*C1q and tumour necrosis factor related protein 5*	−
8407	*TAGLN2*	−3.67	−3.33	−3.50	*Transgelin 2*	+
27230	*SERP1*	−3.14	−3.45	−3.30	*Stress-associated endoplasmic reticulum protein 1*	+
359845	*FAM101B*	−3.35	−2.87	−3.11	*Family with sequence similarity 101, member B*	+
5756	*TWF1*	−3.03	−2.84	−2.93	*Twinfilin, actin-binding protein, homologue 1 (Drosophila)*	+
79794	*C12orf49*	−3.04	−2.69	−2.86	*Chromosome 12 open reading frame 49*	+
2697	*GJA1*	−2.17	−3.07	−2.62	*Gap junction protein, α 1, 43 kDa*	+
4860	*PNP*	−2.65	−2.54	−2.60	*Purine nucleoside phosphorylase*	+
57580	*PREX1*	−2.07	−2.44	−2.26	*Phosphatidylinositol-3,4,5-trisphosphate-dependent Rac exchange factor 1*	+
8683	*SFRS9*	−2.33	−2.14	−2.24	*Splicing factor, arginine/serine-rich 9*	+
23531	*MMD*	−2.37	−2.08	−2.22	*Monocyte to macrophage differentiation-associated*	+
2539	*G6PD*	−2.31	−2.06	−2.18	*Glucose-6-phosphate dehydrogenase*	+
84912	*SLC35B4*	−2.31	−2.05	−2.18	*Solute carrier family 35, member B4*	+
7117	*TMSL3*	−2.06	−2.30	−2.18	*Thymosin-like 3*	+
10487	*CAP1*	−2.25	−2.08	−2.17	*CAP, adenylate cyclase-associated protein 1 (yeast)*	+
5757	*PTMA*	−2.25	−2.04	−2.15	*Prothymosin, α*	+
378	*ARF4*	−2.12	−2.14	−2.13	*ADP-ribosylation factor 4*	+

**Table 3:** Relationships between TAGLN2 expression and clinicopathological factors in tissue microarray

		**TAGLN2 expression (score)**			
**Characteristics**	***n***	**Low (0–2)**	**Moderate (3 to 4)**	**High (5 to 6)**	***P*-values**
*Normal and BC tissue*					
Normal	8	8	0	0	0.0202
Cancer	47	22	20	5	
					
*Age at presentation*					
>65 years	17	9	6	2	0.7499
⩽65 years	30	13	14	3	
					
*Sex*					
Male	38	17	18	3	0.2635
Female	9	5	2	2	
					
*Histological grade*					
G1+G2	28	14	14	0	0.0148
G3	19	6	8	5	
					
*Tumour status*					
T1	10	5	4	1	0.9536
T2+T3+T4	28	15	11	2	
Unknown	9				
					
*LN metastasis*					
N0	40	50	17	3	0.5195
N1	1	0	1	0	
Unknown	6				
*Distant metastasis*					
M0	43	22	18	3	0.0145
M1	4	0	2	2	

Abbreviations: TAGLN2=transgelin 2; BC=bladder cancer; LN=lymph node.
