# Supplementary material for: The tumour-suppressive function of miR-1 and miR-133a targeting TAGLN2 in bladder cancer
Source: Br J Cancer. 2011 Feb 8;104(5):808–18. doi: 10.1038/bjc.2011.23 (PMC3048214; doi:10.1038/bjc.2011.23)
Supplement: Supplementary Figure Legend [file bjc201123x2.doc]

Supplementary Figure Legend

Cell viability inhibitions in the microRNAs transfectants. Cell proliferation in each transfectant was determined by XTT assay. &ast; &ast;P &lt; 0.0001.
