# Supplementary material for: The tumour-suppressive function of miR-1 and miR-133a targeting TAGLN2 in bladder cancer
Source: Br J Cancer. 2011 Feb 8;104(5):808–18. doi: 10.1038/bjc.2011.23 (PMC3048214; doi:10.1038/bjc.2011.23)
Supplement: Supplementary Table 1 [file bjc201123x3.doc]

**Supplimental Table 1** Patient characteristics

| LDA | | | | |  | qRT-PCR | | | | | |
| --- | --- | --- | --- | --- | --- | --- | --- | --- | --- | --- | --- |
| **Bladder cancer (BC)** | |  |  | |  | **Bladder cancer (BC)** | |  |  | | |
|  | Total number | 11 |  | |  |  | Total number | 23 |  | | |
|  | Median age (range) | 70.4 (56–79) | | |  |  | Median age (range) | 73.0 (56–88) | | | |
|  | Gender |  |  | |  |  | Gender |  |  | | |
|  | Male | 8 |  | |  |  | Male | 16 |  | | |
|  | Female | 3 |  | |  |  | Female | 7 |  | | |
|  | Stage |  |  | |  |  | Stage |  |  | | |
|  | Superficial (pTa) | 4 |  | |  |  | Superficial (pTa) | 5 |  | | |
|  | Invasive (≥pT1) | 7 |  | |  |  | Invasive (≥pT1) | 18 |  | | |
|  | Grade |  |  | |  |  | Grade |  |  | | |
|  | G1 | 0 |  | |  |  | G1 | 0 |  | | |
|  | G2 | 4 |  | |  |  | G2 | 9 |  | | |
|  | G3 | 7 |  | |  |  | G3 | 14 |  | | |
|  | Operation |  |  | |  |  | Operation |  |  | | |
|  | Cystectomy | 6 |  | |  |  | Cystectomy | 14 |  | | |
|  | TUR-BT | 5 |  | |  |  | TUR-BT | 9 |  | | |
|  |  |  |  | |  |  |  |  |  | | |
| **Normal bladder epithelium (NBE)** | | |  | |  | **Normal bladder epithelium (NBE)** | | |  | | |
|  | Total number | 5 |  | |  |  | Total number | 10 |  | | |
|  | Median age (range) | 59.8 (45–73) | | |  |  | Median age (range) | 64.3 (56–75) | | | |
|  | Gender |  |  | |  |  | Gender |  |  | | |
|  | Male | 5 |  | |  |  | Male | 10 |  | | |
|  | Female | 0 |  | |  |  | Female | 0 |  | | |
|  |  |  |  |  |  |  |  |  | |  |  |

LDA: low density microRNA array, TUR-BT: transurethral resection of bladder tumour
