# Supplementary material for: The tumour-suppressive function of miR-1 and miR-133a targeting TAGLN2 in bladder cancer
Source: Br J Cancer. 2011 Feb 8;104(5):808–18. doi: 10.1038/bjc.2011.23 (PMC3048214; doi:10.1038/bjc.2011.23)
Supplement: Supplementary Table 2 [file bjc201123x4.doc]

**Supplimental Table 2** construct vector insert sequence

| Target sites | 3'UTR position | insert sequence |
| --- | --- | --- |
| *miR-1* | 71–77 | atattttagcagtgacattcccagagagccccagagctct |
|  | 185–191 | tcccccatgcttactaatacattcccttccccatagccat |
|  | 348–354 | ctgagctctgtgtcctccgttcattccatggctgggagtc |
| *miR-133a* | 214–220 | ccatagccatcaaaactggaccaactggcctcttcctttc |
|  | 242–248 | tcttcctttcccctgggaccaaaatttaggggcctcagtc |
