# Supplementary material for: The tumour-suppressive function of miR-1 and miR-133a targeting TAGLN2 in bladder cancer
Source: Br J Cancer. 2011 Feb 8;104(5):808–18. doi: 10.1038/bjc.2011.23 (PMC3048214; doi:10.1038/bjc.2011.23)
Supplement: Supplementary Table 3 [file bjc201123x5.doc]

**Supplimental Table 3**  Down-regulated microRNAs normalized by *RNU48* in BC

|  |  |  |  | Fold Change |
| --- | --- | --- | --- | --- |
| microRNA | p-value | Normal | Cancer | Cancer/Normal |
| hsa-miR-139-3p | 1.57E-02 | 4.73E-04 | 8.45E-06 | 1.79E-02 |
| hsa-miR-133a | 3.50E-02 | 1.17E-01 | 2.48E-03 | 2.12E-02 |
| hsa-miR-383 | 3.60E-02 | 2.74E-03 | 5.85E-05 | 2.14E-02 |
| hsa-miR-204 | 4.50E-02 | 4.51E-03 | 2.08E-04 | 4.61E-02 |
| hsa-miR-1 | 9.40E-03 | 1.52E-03 | 7.16E-05 | 4.72E-02 |
| hsa-miR-139-5p | 1.71E-04 | 8.31E-02 | 4.65E-03 | 5.60E-02 |
| hsa-miR-145 | 2.79E-02 | 6.84E-01 | 4.36E-02 | 6.38E-02 |
| hsa-miR-133b | 3.60E-02 | 1.78E-03 | 1.26E-04 | 7.06E-02 |
| hsa-miR-370 | 2.37E-02 | 8.95E-04 | 8.26E-05 | 9.23E-02 |
| hsa-miR-574-3p | 6.11E-04 | 3.25E-01 | 3.63E-02 | 1.12E-01 |
| hsa-miR-376c | 2.57E-03 | 1.44E-02 | 1.91E-03 | 1.32E-01 |
| hsa-miR-199b-5p | 1.59E-02 | 6.05E-04 | 8.23E-05 | 1.36E-01 |
| hsa-miR-486-3p | 2.74E-02 | 2.89E-03 | 4.00E-04 | 1.38E-01 |
| hsa-miR-214 | 2.62E-03 | 5.08E-02 | 7.29E-03 | 1.43E-01 |
| hsa-let-7c | 1.34E-03 | 4.70E-03 | 7.16E-04 | 1.52E-01 |
| hsa-miR-433 | 4.21E-02 | 6.08E-04 | 9.49E-05 | 1.56E-01 |
| hsa-miR-140-3p | 6.29E-03 | 1.73E-02 | 2.96E-03 | 1.72E-01 |
| hsa-miR-134 | 7.02E-04 | 3.54E-03 | 6.69E-04 | 1.89E-01 |
| hsa-miR-299-5p | 3.99E-02 | 2.19E-04 | 4.22E-05 | 1.92E-01 |
| hsa-miR-493 | 2.83E-02 | 3.28E-04 | 7.15E-05 | 2.18E-01 |
| hsa-miR-143 | 2.14E-02 | 2.60E-01 | 5.83E-02 | 2.24E-01 |
| hsa-miR-411 | 1.43E-03 | 4.58E-03 | 1.05E-03 | 2.29E-01 |
| hsa-miR-218 | 1.83E-03 | 1.66E-02 | 4.06E-03 | 2.44E-01 |
| hsa-miR-196b | 6.14E-03 | 2.76E-02 | 7.56E-03 | 2.74E-01 |
| hsa-miR-126 | 1.08E-03 | 1.87E+00 | 5.50E-01 | 2.95E-01 |
| hsa-miR-199a-5p | 3.69E-02 | 6.06E-04 | 1.81E-04 | 2.98E-01 |
| hsa-miR-379 | 2.05E-02 | 2.05E-03 | 6.32E-04 | 3.08E-01 |
| hsa-miR-195 | 4.40E-02 | 5.54E-02 | 1.74E-02 | 3.14E-01 |
| hsa-miR-376a | 1.01E-02 | 1.10E-03 | 3.56E-04 | 3.24E-01 |
| hsa-miR-186 | 8.04E-04 | 8.68E-02 | 3.10E-02 | 3.57E-01 |
| hsa-miR-99a* | 3.65E-02 | 1.39E-03 | 5.06E-04 | 3.64E-01 |
| hsa-miR-197 | 3.87E-02 | 6.25E-03 | 2.28E-03 | 3.64E-01 |
| hsa-miR-125a-5p | 1.05E-02 | 2.37E-02 | 9.15E-03 | 3.86E-01 |
| hsa-miR-320 | 3.81E-02 | 2.34E-01 | 9.70E-02 | 4.14E-01 |
| hsa-miR-628-5p | 2.48E-03 | 1.52E-03 | 6.39E-04 | 4.22E-01 |
| hsa-miR-342-3p | 4.76E-03 | 8.39E-02 | 3.61E-02 | 4.30E-01 |
| hsa-miR-152 | 2.23E-02 | 1.42E-02 | 6.51E-03 | 4.60E-01 |
| hsa-miR-28-3p | 1.08E-02 | 6.33E-02 | 3.15E-02 | 4.98E-01 |
| hsa-miR-193b | 3.84E-03 | 1.48E-01 | 7.75E-02 | 5.26E-01 |
| hsa-miR-30c-2* | 3.32E-02 | 7.20E-06 | 3.99E-06 | 5.54E-01 |
| hsa-miR-484 | 2.31E-02 | 2.18E-01 | 1.31E-01 | 6.01E-01 |
