# Supplementary material for: The tumour-suppressive function of miR-1 and miR-133a targeting TAGLN2 in bladder cancer
Source: Br J Cancer. 2011 Feb 8;104(5):808–18. doi: 10.1038/bjc.2011.23 (PMC3048214; doi:10.1038/bjc.2011.23)
Supplement: Supplementary Table 4 [file bjc201123x6.doc]

**Supplimental Table 4** Down-regulated microRNAs normalized by *MammU6* in BC

|  |  |  |  | Fold Change |
| --- | --- | --- | --- | --- |
| microRNA | p-value | Normal | Cancer | Cancer/Normal |
| hsa-miR-133a | 1.71E-02 | 8.87E-03 | 3.29E-04 | 3.71E-02 |
| hsa-miR-204 | 5.13E-03 | 3.29E-04 | 1.89E-05 | 5.73E-02 |
| hsa-miR-1 | 1.04E-02 | 1.21E-04 | 9.27E-06 | 7.68E-02 |
| hsa-miR-139-5p | 8.50E-03 | 7.11E-03 | 5.85E-04 | 8.23E-02 |
| hsa-miR-370 | 2.17E-04 | 6.19E-05 | 7.67E-06 | 1.24E-01 |
| hsa-miR-133b | 3.79E-03 | 1.28E-04 | 1.76E-05 | 1.38E-01 |
| hsa-miR-574-3p | 6.01E-03 | 2.71E-02 | 3.89E-03 | 1.43E-01 |
| hsa-miR-376c | 2.85E-02 | 1.25E-03 | 1.87E-04 | 1.50E-01 |
| hsa-miR-214 | 7.06E-03 | 4.22E-03 | 8.20E-04 | 1.94E-01 |
| hsa-let-7c | 2.23E-03 | 3.78E-04 | 7.55E-05 | 2.00E-01 |
| hsa-miR-140-3p | 1.34E-02 | 1.49E-03 | 3.13E-04 | 2.10E-01 |
| hsa-miR-134 | 7.55E-03 | 3.02E-04 | 6.43E-05 | 2.13E-01 |
| hsa-miR-411 | 5.23E-03 | 3.71E-04 | 1.14E-04 | 3.07E-01 |
| hsa-miR-218 | 4.18E-02 | 1.44E-03 | 4.42E-04 | 3.08E-01 |
| hsa-miR-196b | 9.56E-03 | 2.25E-03 | 7.51E-04 | 3.33E-01 |
| hsa-miR-199a | 1.96E-02 | 4.78E-05 | 1.67E-05 | 3.49E-01 |
| hsa-miR-885-5p | 1.38E-02 | 2.92E-05 | 1.07E-05 | 3.68E-01 |
| hsa-miR-186 | 3.64E-02 | 7.37E-03 | 3.18E-03 | 4.32E-01 |
| hsa-miR-320 | 3.13E-02 | 1.82E-02 | 9.82E-03 | 5.40E-01 |
